# Identifying a Window of Vulnerability during Fetal Development in a Maternal Iron Restriction Model

**DOI:** 10.1371/journal.pone.0017483

**Published:** 2011-03-15

**Authors:** Camelia Mihaila, Jordan Schramm, Frederick G. Strathmann, Dawn L. Lee, Robert M. Gelein, Anne E. Luebke, Margot Mayer-Pröschel

**Affiliations:** 1 Department of Biomedical Genetics, University of Rochester, Rochester, New York, United States of America; 2 Department of Neurobiology and Anatomy, University of Rochester, Rochester, New York, United States of America; 3 Department of Biomedical Engineering, University of Rochester, Rochester, New York, United States of America; 4 Department of Pathology and Laboratory Medicine, University of Rochester, Rochester, New York, United States of America; 5 Department of Environmental Medicine, University of Rochester, Rochester, New York, United States of America; University of Queensland, Australia

## Abstract

It is well acknowledged from observations in humans that iron deficiency during pregnancy can be associated with a number of developmental problems in the newborn and developing child. Due to the obvious limitations of human studies, the stage during gestation at which maternal iron deficiency causes an apparent impairment in the offspring remains elusive. In order to begin to understand the time window(s) during pregnancy that is/are especially susceptible to suboptimal iron levels, which may result in negative effects on the development of the fetus, we developed a rat model in which we were able to manipulate and monitor the dietary iron intake during specific stages of pregnancy and analyzed the developing fetuses. We established four different dietary-feeding protocols that were designed to render the fetuses iron deficient at different gestational stages. Based on a functional analysis that employed Auditory Brainstem Response measurements, we found that maternal iron restriction initiated prior to conception and during the first trimester were associated with profound changes in the developing fetus compared to iron restriction initiated later in pregnancy. We also showed that the presence of iron deficiency anemia, low body weight, and changes in core body temperature were not defining factors in the establishment of neural impairment in the rodent offspring.

Our data may have significant relevance for understanding the impact of suboptimal iron levels during pregnancy not only on the mother but also on the developing fetus and hence might lead to a more informed timing of iron supplementation during pregnancy.

## Introduction

The clinical importance and prevalence of iron deficiency (ID) make the understanding of this micronutrient deficiency an important challenge for both the scientific and medical communities. Iron is an essential micronutrient and ID affects more than 2 billion people around the world. ID occurs in many forms ranging from marginal tissue iron depletion to the most severe form of iron deficiency anemia (IDA). It has been estimated that, globally, 50% of anemia can be attributed to ID. IDA ranks at number 9 among 26 mortality risk factors and accounts for over 800,000 deaths and 35 million disability-adjusted lost life years [1]. North America alone bears 1.4% of the global burden of ID and IDA and it has been estimated that 35–58% of healthy women show some degree of ID, with a higher prevalence during pregnancy [1], [2], [3], [4], [5]. This high prevalence of ID during pregnancy seems at odds with the practice of routine iron supplementation as part of the prenatal care provided in most developed countries. Factors that contribute to the still high prevalence are complex and include concerns of early iron supplementation generating oxidative stress [6], a low compliance rate (50%) of taking iron supplements even with optimal motivation and guidance due to the undesirable adverse effects [7], [8], [9], and a rise in risk factors like type-2 diabetes and obesity that can cause ID and IDA despite adequate food intake [10], [11], [12], [13].

ID and untreated IDA during pregnancy have many negative consequences for the offspring and have been shown to be associated with a higher incidence of low birth weight and prematurity [14], [15], [16], [17], [18], long-term cognitive abnormalities such as language learning impairments and behavioral changes [19], [20], [21], [22], [23], [24], [25], [26], [27], [28], [29], [30], [31], [32], [33], [34], [35], alteration in thermoregulation [36], changes in lipid metabolism [37], stroke and seizures [38], [39], altered motor function and coordination [40], [41], [42], and in many cases alteration of Auditory Brainstem Responses (ABRs, [35], [43], [44], [45], [46], a measure of nerve impulse conduction in the auditory system). Gestational ID also has been shown to change iron homeostasis in the offspring resulting in increased risk of developing ID later in life despite adequate nutrition [47], [48], [49]. It is alarming that the reported prevalence of ID for US children under 2 years of age is estimated to be 25% [23], [50], [51], [52], which might well be an underestimation due to the difficulty in diagnosing ID in the absence of anemia.

Despite the recognition that ID during pregnancy can have multiple adverse effects on the developing fetus, it remains elusive to what degree and during which gestational time window maternal ID has to occur to affect fetal development to a degree that leads to functionally relevant long-term impairments.

We addressed this question using a highly controlled animal model system that allowed us to stage the initiation of ID during pregnancy using a defined feeding regimen and to analyze the fetal development as well as the neural function in the young adult offspring. Our prior studies on the effects of IDA during pregnancy suggested that IDA affects a very early arising precursor cell pool. The impairment of this cellular population is likely to contribute to an ultimate disruption of proper CNS development during postnatal development [53]. Based on those studies, we now address the fundamental questions of whether (i) the generation of neural impairment in the offspring is limited to cases of maternal IDA as opposed to ID without anemia and whether (ii) the time window during which the iron depletion occurs in the dam determines the degree of functional impairments in the offspring.

## Results

### Maternal iron restriction that does not cause severe maternal anemia renders the embryo iron depleted with postnatal onset of anemia

**Figure 1:**
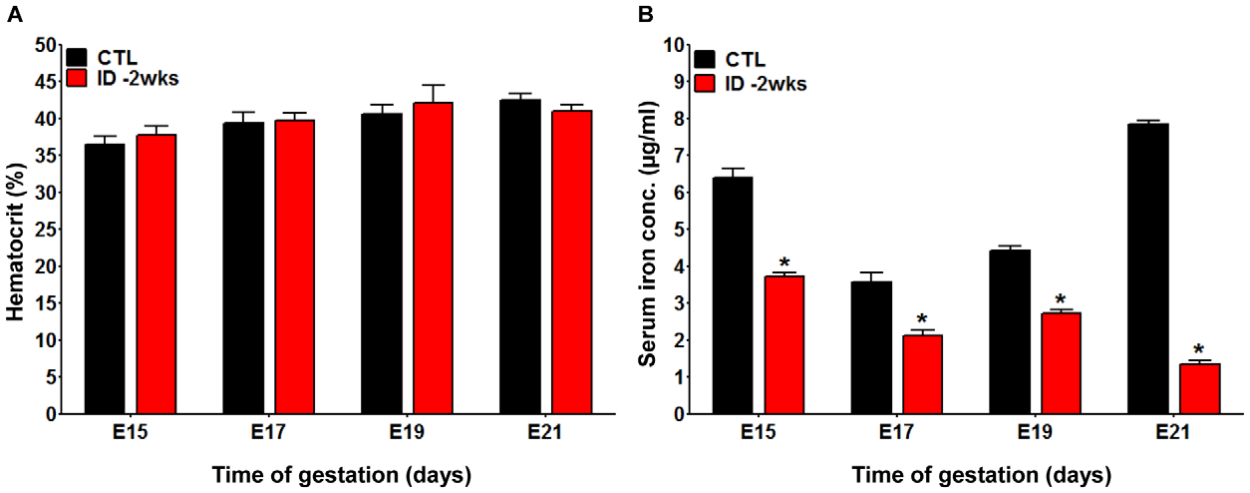
Iron restriction two weeks before conception induces maternal iron deficiency without severe anemia. A. Hematocrit levels of pregnant rats exposed to an iron-deficient diet two weeks prior to conception (*ID -2wks*) (red bars) were evaluated at gestational day E15, E17, E19, and E21 and compared to time matched pregnant rats that received a control diet (black bars). *CTL* pregnant dams: n = 6 for each gestational age; *ID -2wks* pregnant dams: n = 6 for each gestational age; expressed values are mean ± SEM. **B.** The serum iron concentrations in iron-deficient diet treated animals (*ID -2wks*) and control animals (*CTL*) were determined via atomic absorption spectroscopy (AAS) at E15, E17, E19, and E21 (*CTL* pregnant rats: n = 3 for each time point; *ID -2wks* pregnant rats: n = 3 for each time point; Mean ± SEM, * p<0.001). While there were no significant changes in the hematocrit values between the two groups, iron restricted dams showed significantly lower serum iron concentrations compared to control dams, confirming their iron deficiency (ID) status.

One of the major challenges in understanding the impact of iron on development is the difficulty of measuring iron concentrations in the developing embryo. Animal models allow us to gain insight into the relationship between maternal iron intake and fetal iron levels. We established a feeding protocol in which rat dams were provided a customized iron-deficient diet that led to maternal iron deficiency (ID) but not to severe iron deficiency anemia (IDA). As shown in **Figure 1A**, this diet did not generate a discernable difference in hematocrit values in the pregnant dams compared to control dams, although hemoglobin (Hb) and red blood cell counts (RBC) were slightly below the control range by 21 days of gestation. Even though severe anemia was absent in the dams, we found progressively reduced serum iron levels, ranging from a >40% reduction at 15 days of gestation up to an 82% decrease at 21 days of gestation, relative to controls (CTLs; **Figure 1B**).

**Figure 2:**
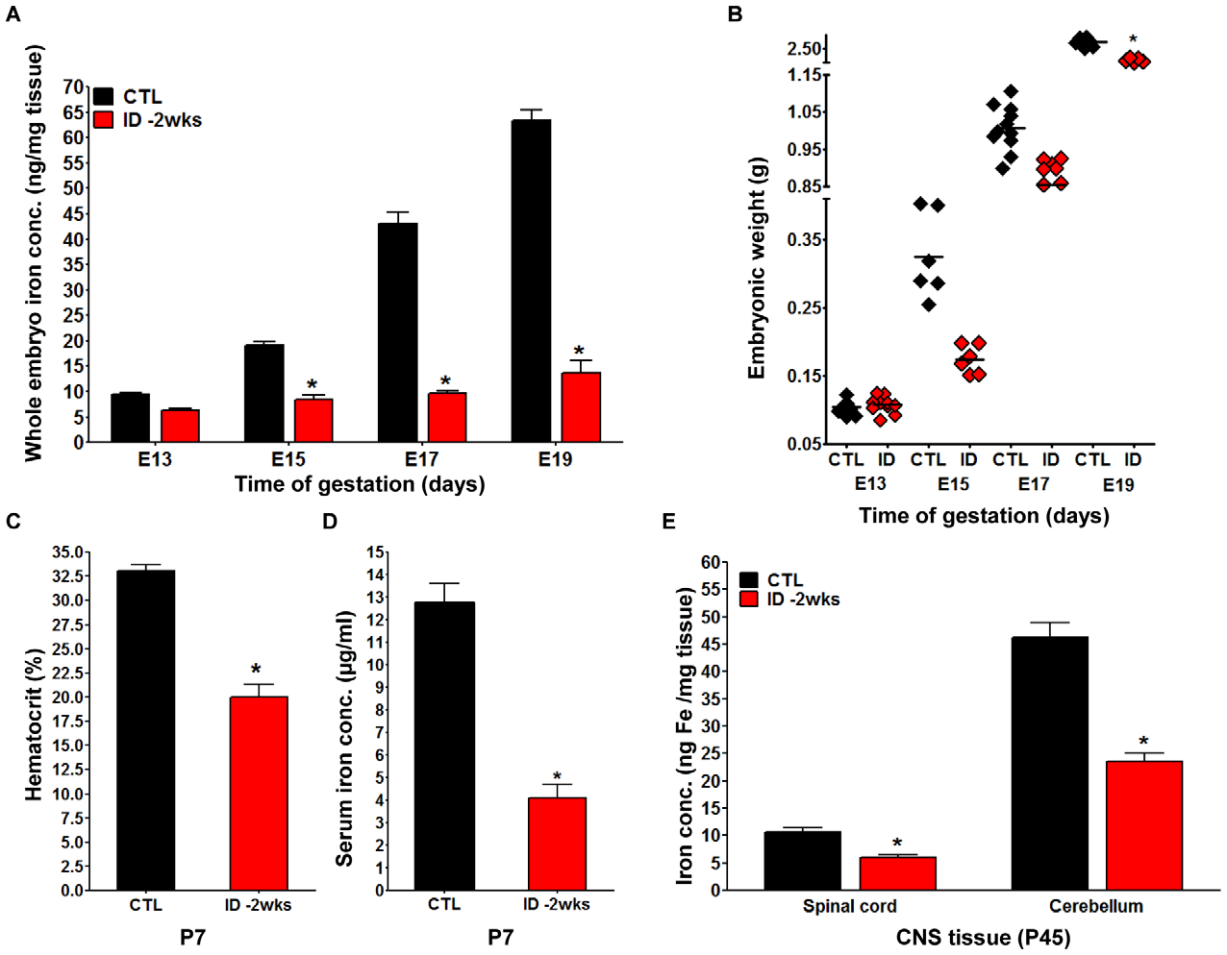
Maternal iron restriction prior to conception is associated with embryonic iron deficiency, postnatal anemia, and reduced postnatal central nervous system iron levels. The *ID -2wks* and *CTL* pregnant dams were sacrificed at different time points during gestation (E13, E15, E17, and E19) and embryos were characterized in respect to total iron content by AAS analysis. **A**. The nutritional *ID -2wks* regimen generated embryonic ID as early as E15 (Mean ± SEM, * p<0.001). **B**. The embryos of *ID -2wks* dams were significantly smaller than *CTL* embryos at all gestational ages after E13. (For A and B: at E13, n = 12 *CTL* embryos and n = 10 *ID-2wks* embryos; at E15, n = 6 *CTL* embryos and n = 6 *ID -2wks* embryos; at E17, n = 11 *CTL* embryos and n = 13 *ID -2wks* embryos; at E19, n = 6 *CTL* embryos and n = 6 ID *-2wks* embryos; for all gestational ages, each *CTL* and *ID -2wks* group was composed of embryos from 3 separate litters; Mean ± SEM, * p<0.05). **C**. Hematocrit measurements in *ID -2wks* pups (red bars) revealed values significantly lower than in *CTL* pups (black bars) at postnatal day 7 (P7) (Mean ± SEM, * p<0.0001). **D**. Serum iron concentrations measured at P7 were significantly decreased in blood samples collected from *ID -2wks* rat pups (red bars) compared to *CTL* pups (black bars)(For C and D: *CTL* and *ID-2wks* each had n = 8 pups from 3 separate litters, Mean ± SEM, * p<0.0001). **E**. AAS at postnatal day 45 showed reduced iron level in ID spinal cord and cerebellum compared to CTL samples (spinal cord: n = 8 *CTL* and n = 5 *ID -2wks* samples; cerebellum: n = 5 *CTL* and n = 5 *ID -2wks* samples; each *CTL* and *ID -2wks* group contained tissue samples harvested from 3 separate litters, Mean +/− SEM, * p<0.05).

To determine the impact of the decrease in serum iron concentration in the dam on the iron status in the developing embryo, we measured the iron concentration of whole embryos using atomic absorption spectroscopy (AAS; **Figure 2A** **)**. While we did not see a significant reduction in tissue iron concentrations at E13, at E15 embryos from iron deficient dams had only 44% of the levels of iron seen in control embryos. Despite a developmental increase in total iron concentration in the iron deficient embryos from an average of 8 ng Fe/mg tissue at E15 to approximately 14 ng Fe/mg at E19, these values were far below the normal Fe concentrations that increased from 19 ng Fe/mg at E15 up to 63 ng Fe/ml at E19. This lack of iron accumulation in the embryos was correlated with the respective total body weights. At E13, when the embryos did not yet show a significant difference in Fe concentration compared to controls, there also was not a difference in the weight of the embryos. At E15, E17, and E19, however, there was a persistent lack of weight gain in the iron deficient embryos compared to controls, rendering ID fetuses nearly 40% lighter than control fetuses at E15. By E19, the weight increased although ID embryos never reached the same weight as controls **(** **Figure 2B** **)**.

In order to determine whether a continuation of the maternal feeding protocol postpartum has a persistent impact on the developing pups, we maintained the dams on the diet they received throughout gestation and tested the iron status of the pups one week after birth. As expected, the iron status of the pups continued to deteriorate and after one week serum iron concentrations were reduced by 63% and hematocrit levels showed a 40% decrease (**Figure 2C, D**). While the reduction of iron concentrations in whole embryos and in the serum of postnatal animals suggested a change in overall iron homeostasis, our functional readout was focused on the impact of iron deficiency on brain development. We were, therefore, particularly interested in the status of brain iron levels in postnatal animals. AAS analysis of two central nervous system (CNS) regions that are particularly easy to harvest and allow for a good volumetric normalization (spinal cord and cerebellum) confirmed that there was a significant decrease in the concentration of iron in these tissues (**Figure 2E**). The decrease in iron concentration was more pronounced in the cerebellum, which had a much higher base-line level than the spinal cord.

### Embryonic iron deficiency leads to changes in auditory nerve conduction velocity

Having shown that the early maternal ID severely impairs the iron homeostasis and weight of the offspring, we next asked whether this gestational insult also results in impaired CNS development in the offspring. Studies in humans, monkeys, and rodent animal models have suggested functional impairments in neural signaling as measured by impaired conduction velocity in the auditory system. This measurement of nerve conductivity is particularly useful as it allows for a unified and non-invasive analysis of a stereotypical neural response profile that results in a wave diagram with each wave representing the neural activity along the ascending pathway (see for review [54], [55], [56], [57]). Specific ABR wave parameters like interpeak latencies between P2 and P1 are thought to be a reflection of the myelination status [58], [59], while changes in P3 and P4 interpeak latencies are also suggestive of impairments in synapse maturation [60], [61], [62], [63]. Measurements of Distortion-Product of Otoacoustic Emissions (DPOAE) can be used to exclude impairments that are due to a peripheral loss of hair cell function that would also affect the general neural output [64], [65], [66], [67].

**Figure 3:**
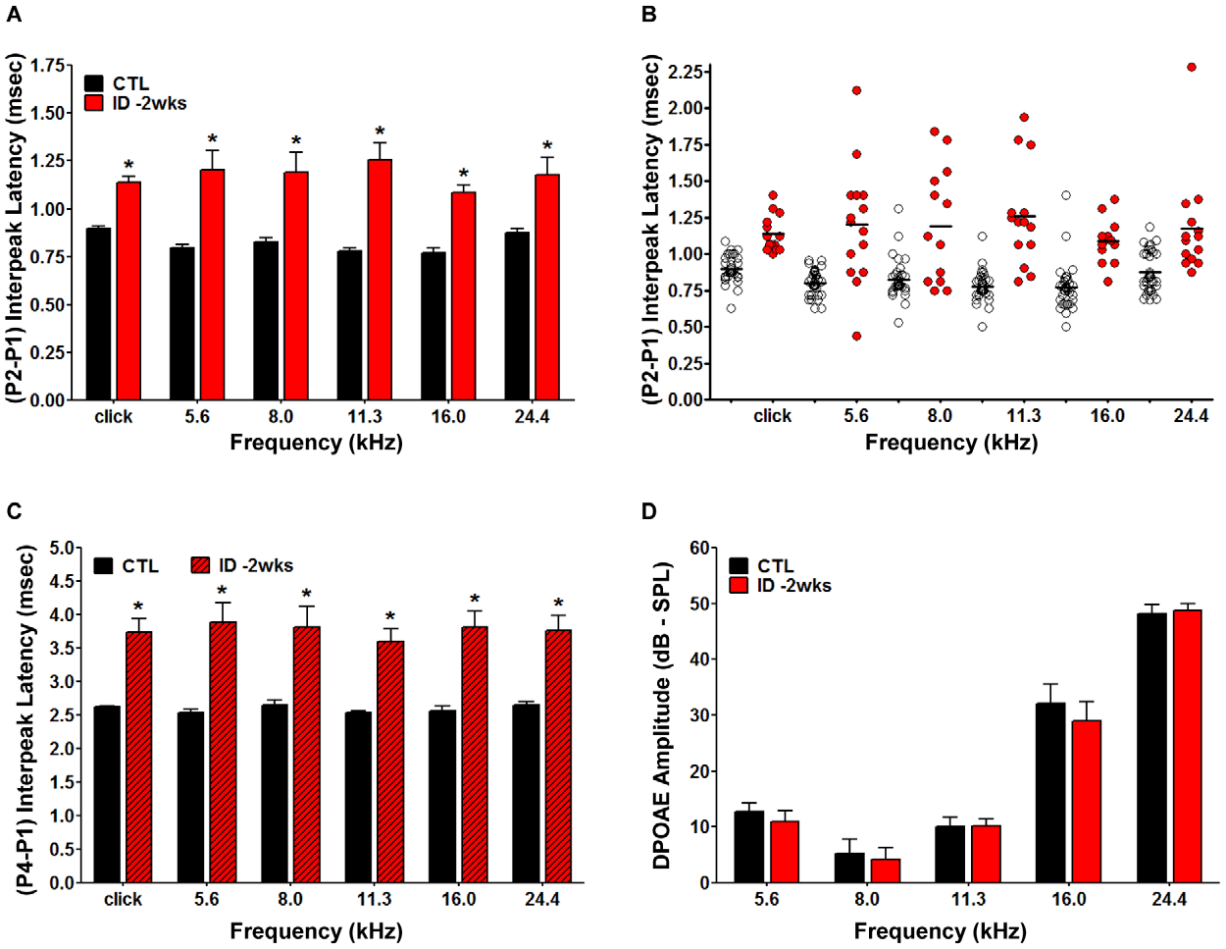
Auditory Brainstem Responses (ABR) measurement revealed decreased conduction velocities in the offspring born to iron deficient dams. Female Sprague-Dawley rats were provided with the iron-deficient diet two weeks before conception and remained on this diet throughout pregnancy and lactation. Rat pups born to these dams were maintained on iron-deficient diets until testing at postnatal day 45 (P45). Cochlear function was also assessed for all animals (*CTL* and *ID -2wks* groups) at ∼P45. **A**. The ABR (P2-P1) interpeak latencies recorded from *ID -2wks* (red bars) animals showed a highly significant increase compared to *CTL* (black bars) at all frequencies tested. Auditory nerve function was analyzed by measuring the Auditory Brainstem Responses (ABR) to tone pips at 5 log-spaced frequencies from 5.6 to 24.4 kHz (at 70 dB sound pressure level-SPL). ABR peaks and troughs were identified using a semi-automated tool and verified by a trained observer. Latencies were calculated from onset of stimulus and the (P2-P1) interpeak latency was computed; Mean ± SEM, p<0.0001). **B**. The ABR interpeak latencies displayed increased variability in the *ID -2wks* animals (red circles) compared to *CTL* (open circles) and the mean value was increased in iron deficient animals. **C**. The ABR (P4-P1) interpeak latencies recorded from *ID -2wks* (red hatched bars) animals were significantly prolonged compared to *CTL* (black bars) at all frequencies tested (Mean ± SEM, p<0.0001). **D**. The ID dietary regimen was not associated with outer hair cell damage or cochlear dysfunction as revealed by Distortion-Product of Otoacoustic Emissions (DPOAE) analysis. There was no significant difference between control (black bars) and iron-deficient rats (red bars) in DPOAE amplitudes. (For A–D: n = 35 *CTL* rats from 5 separate litters and n = 15 *ID -2wks* rats from 3 separate litters; Mean ± SEM).

To determine the impact of the early maternal ID on the CNS development of the offspring, we conducted an ABR analysis of the ID and normal offspring at P40–45 days, a time point at which the auditory system is fully developed. As shown in **Figure 3A**, ABR latency analysis revealed significantly increased P2-P1 interpeak latencies in ID offspring compared to CTLs at all frequencies examined, ranging from 0.25±0.18 ms to 0.49±0.034 ms (* p<0.0001). When we compared the interpeak latency values of the individual animals in the ID group to control animals, it was apparent that iron deficient animals showed a more pronounced variability across all animals compared to controls (**Figure 3B**). We also analyzed the interpeak latency of P4-P1 to determine whether the impairment affects other aspects of auditory information processes detectable by ABR analysis. As shown in **Figure 3C**, interpeak latencies P4-P1 were also significantly increased in the ID group. In order to exclude possible defects of inner and outer hair cells that would influence the ABR measurements, we also conducted a DPOAE assessment. As shown in **Figure 3D**, DPOAE amplitude values of ID group were indistinguishable from the control group at all frequencies which strongly suggested normal hair cell function. Wave morphology was not different compared to CTL and hearing thresholds were not elevated in ID animals (data not shown). Taken together, our data show that animals exposed to ID during gestation develop an IDA that is associated with a significant defect in neural function as determined by ABR latency analysis.

### Delayed onset of gestational iron restriction does not prevent the development of IDA in the offspring

**Figure 4:**
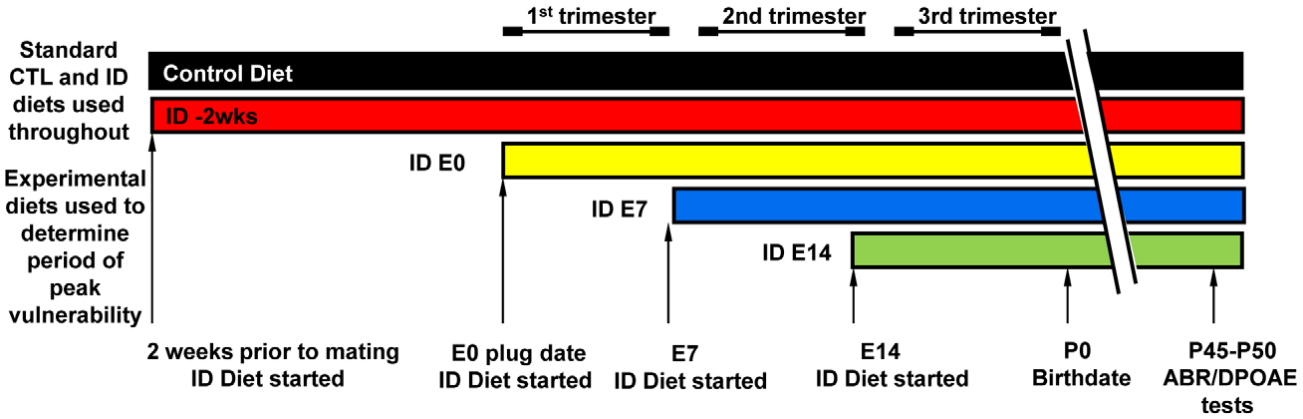
Experimental design of the dietary regimens. To determine the restricted developmental window in which maternal iron restriction impacts fetal development, we established one control (CTL) group and four experimental groups of iron deficient animals: ***CTL*** group: animals received control iron diet (240 µg Fe/g food) throughout the experiment; ***ID*** *-* ***2wks*** group: animals began the iron-deficient diet (2–6 µg Fe/g food) two weeks before conception; ***ID E0*** group***:*** the iron-deficient diet was introduced the day of plug detection (E0); ***ID E7*** group**:** animals were placed on the iron-deficient diet on embryonic day 7 (E7); ***ID E14*** group**:** animals were fed the iron-deficient diet starting on embryonic day 14 (E14); All pregnant Sprague–Dawley female rats in different diet regimens groups were maintained on the respective diet from the assigned start point throughout pregnancy and lactation. After weaning, the pups were maintained on iron-deficient diet until the day of auditory testing (postnatal day 45).

The dietary regimen used so far in our study (*ID -2wks*) was selected to model a pre-pregnancy state that is defined by suboptimal tissue iron concentrations of the dam prior to conception that does not result in a stage of severe maternal IDA by the time the pups are born. While this dietary regimen led to neural impairment in the offspring, the development of IDA in the offspring, lack of weight gain, and lower core body temperature were all factors that could have contributed to the ABR latency defect. In order to begin to better define which of these factors might play a defining role to the development of neural impairment in the offspring, we changed the onset of the timing of the iron restriction along specific gestational windows. As outlined in **Figure 4**, we generated three additional groups of rats in which the iron-deficient diet (2–6 µg Fe/g) was introduced at the beginning of pregnancy *(ID E0)*, and at the beginning of the 2nd (*ID E7*) or the 3rd (*ID E14*) trimester. All experimental groups remained on the iron-deficient diet during lactation and after weaning until the pups reached postnatal day 45, when the animals were subjected to hematological analysis along with weight and temperature measurements followed by an ABR measurement as outlined before. Control group (*CTL*) consisted of age-matched rats whose dams were maintained on a control diet which contains 240 µg Fe/g and is identical to the iron deficient diet in all other ingredients.

**Figure 5:**
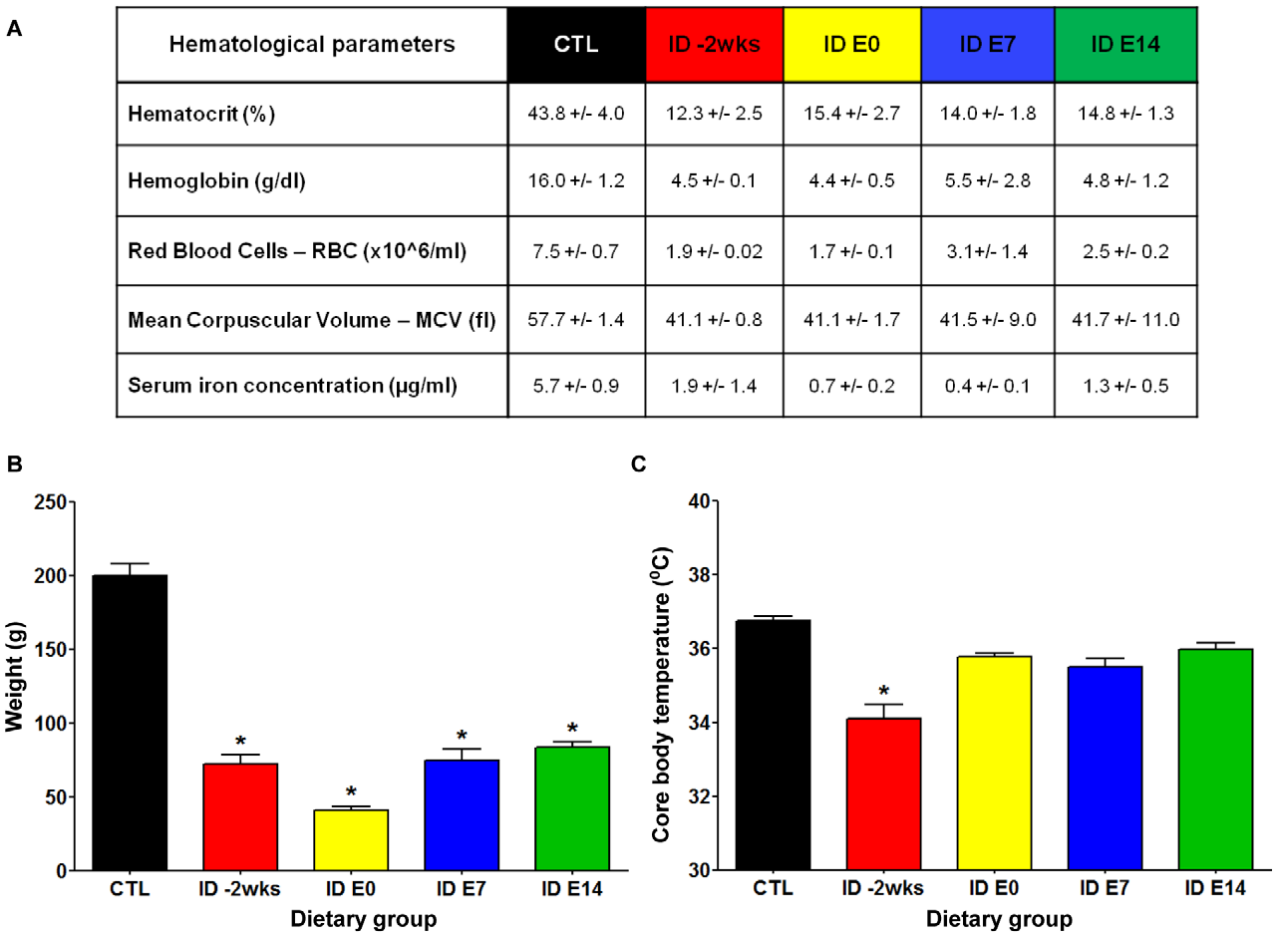
The experimental dietary regimens established iron deficiency anemia (IDA) and caused changes in body weight and core body temperature in the offspring. The hematological status, weight, and core temperature were evaluated in each animal after the ABR test completion on postnatal day 45 in the various dietary groups (depicted in Figure 4). **A.** The table summarizes the results of the hematological tests. All groups of experimental iron deficient animals (*ID -2wks, ID E0*, *ID E7*, and *ID E14*) presented with significantly decreased hematocrit (Ht) values, significantly lower hemoglobin (Hb) levels, profoundly decreased red blood cells (RBC) counts and markedly reduced mean corpuscular volume (MVC) compared to controls, demonstrating the presence of anemia in dietary iron restricted rats. Serum iron concentrations in iron-deficient diet treated animals were significantly lower compared to control animals, confirming the anemic condition of experimental animals. (Ht measurement: n = 11 *CTL* rats, n = 9 *ID -2wks* rats, n = 11 *ID E0* rats, n = 9 *ID E7 rats*, and n =  9 *ID E14 rats*; Hb, RBC, and MCV determination: n = 6 *CTL rats*, n = 4 *ID -2wks* rats, n = 4 *ID E0* rats, n = 4 *ID E7 rats*, and n = 6 *ID E14 rats*; Mean ± SEM, * p<0.0001; serum iron analysis: n = 5 rats for all groups, Mean ± SEM, * p<0.01; each *CTL* and experimental group included rats from 3 separate litters). **B.** Experimental iron deficient animals exhibited marked growth retardation compared with controls. (n = 35 *CTL* rats, n = 15 *ID -2wks* rats, n = 14 *ID E0* rats, n = 27 *ID E7* rats, and n = 29 *ID E14* rats; all CTL and experimental groups contained rat pups from at least 3 separate litters; Mean ± SEM, * p<0.0001). **C.** Experimental group *ID -2wks* (n = 18 rats from 3 separate litters) offspring exhibit significantly reduced core body temperature in comparison to *CTL* animals (n = 17 rats from 3 different litters; * p<0.05). These changes are not seen in the other three experimental groups (n = 13 *ID E0* rats, n = 13 *ID E7* rats, and n = 14 *ID E14* rats). All groups are composed of animals from 3 separate litters.

As shown in **Figure 5A**, the hematological analysis revealed that the offspring from all the dietary groups had established severe anemia by the time of the ABR measurement at P45. All groups showed a significant reduction in hematocrit values, hemoglobin concentrations, red blood cell counts (RBC), mean corpuscular volumes (MCV) and serum iron concentrations relative to the CTL group. Other parameters that could impact neural function were also affected but to various degrees depending on the time when the ID diet was introduced to the pregnant dam. As shown in **Figure 5B**, all iron deficient groups showed a significant decrease in body weight, while only the *ID-2wks* offspring presented with a significant decrease in core body temperature compared to controls. Core body temperatures were not affected when the iron restriction started at embryonic day 0 *(ID E0),* day 7 (*ID E7*) or day 14 (*ID E14)* and were comparable to control values (**Figure 5C**).

### The establishment of a neural impairment in the offspring is dependent on the onset of the iron restriction during pregnancy defining a window of vulnerability

**Figure 6:**
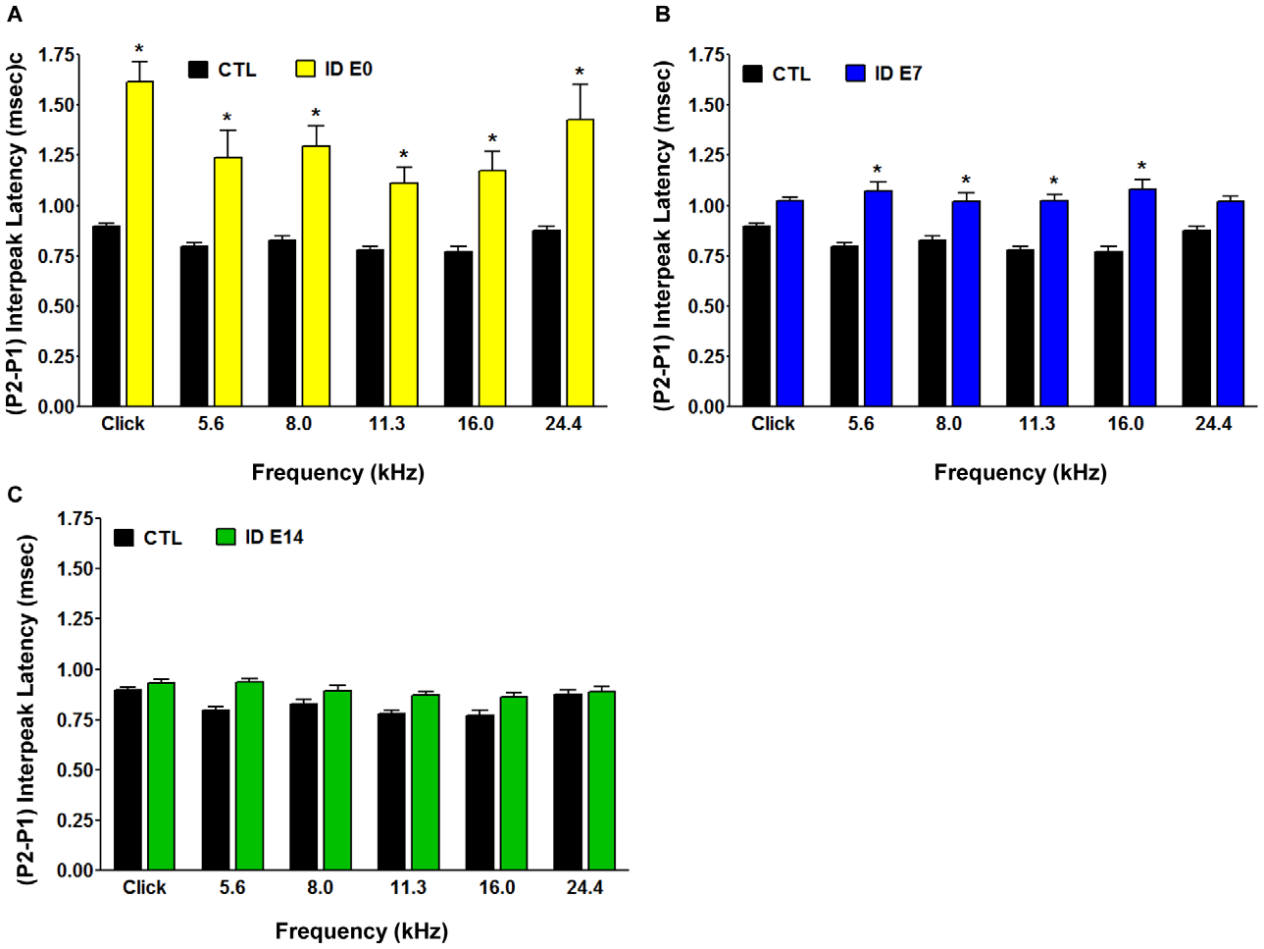
Increased Auditory Brainstem Response (ABR) conduction time in the offspring of various dietary groups can define a specific window of vulnerability. Auditory nerve function was analyzed by measuring the auditory brainstem responses (ABR) to tone pips at 5 log-spaced frequencies from 5.6 to 24.4 kHz (at 70 dB SPL). ABR peaks and troughs were identified using a semi-automated tool and verified by a trained observer. Latencies were calculated from onset of stimulus and the (P2-P1) interpeak latency was computed. The rats were sedated with an intraperitoneal injection of ketamine (80 mg/kg) and acepromazine (1 mg/kg). **A**. The offspring born to dams exposed to ID diet from the time of mating, *ID E0 group* (yellow bars), displayed significantly prolonged ABR (P2-P1) interwave latencies at all frequencies examined compared to controls (*CTL*, black bars) (n = 35 *CTL* rats, n = 14 *ID E0* rats; Mean ± SEM, *p<0.05). **B.** The offspring of dams provided with the iron-deficient diet in the second trimester, *ID E7* (blue bars), exhibited significantly prolonged (P2-P1) interpeak latencies at a subset of frequencies tested compared to *CTL* (black bars) (n = 35 *CTL rats*; n = 27 *ID E7 rats*, *p<0.05). **C.** The pups born to dams provided with the iron-deficient diet in the third trimester of pregnancy, *ID E14* (green bars, n = 29), had normal ABR (P2-P1) interpeak latencies compared to *CTL* (black bars, n = 35). The control and experimental dietary groups were composed of rat pups from 3 separate litters.

To monitor whether the delayed onset of the maternal iron restriction changes the functional neural impairments in the offspring we had detected in the *ID-2wks* dietary group, we performed ABR testing of the P45 offspring as outlined before. In all three groups DPOAE analysis revealed again that outer hair cell function was comparable to control animals (data not shown). ABR tests in the *ID E0* and *ID E7* offspring showed prolonged P2-P1 interpeak latencies ranging from 0.17±0.06 ms up to 0.39±0.05 ms compared to CTL values. ABR latency responses at the highest frequency (24 kHz) did not reach statistically significant levels in the *ID E7* group (**Figure 6A and 6B**). There was also a decreased severity of the ABR defects with a later occurring insult. Surprisingly, when pregnant females were exposed to iron restriction at the beginning of the third trimester (*ID E14*), and the offspring was subjected to ABR testing, we did not find any statistically significant interpeak latency differences in the *ID E14* group compared to the control group across all frequencies (**Figure 6C**).

**Figure 7:**
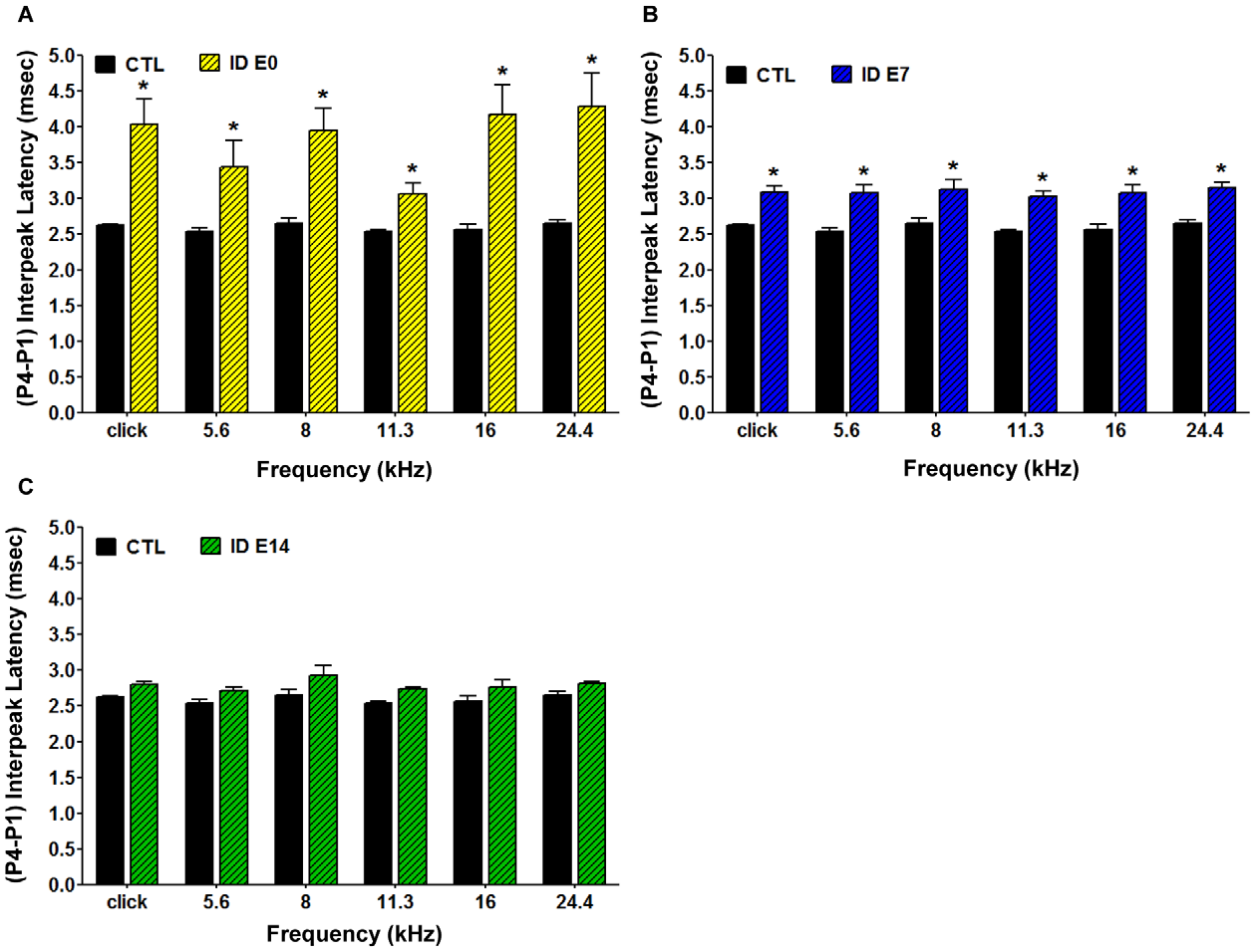
Conduction velocity is decreased throughout the brainstem along the auditory pathway in the offspring of the various dietary groups, as reflected by ABR (P4-P1) interpeak latency. Auditory nerve function was analyzed by measuring the auditory brainstem responses (ABR) to tone pips at 5 log-spaced frequencies from 5.6 to 24.4 kHz (at 70 dB SPL). ABR peaks and troughs were identified using a semi-automated tool and verified by a trained observer. Latencies were calculated from onset of stimulus and the (P4-P1) interpeak latency was computed. The rats were sedated with an intraperitoneal injection of ketamine (80 mg/kg) and acepromazine (1 mg/kg). **A**. The offspring born to dams provided with ID diet from the time of mating, *ID E0 group* (yellow hatched bars), displayed significantly prolonged ABR (P4-P1) interpeak latencies at all frequencies examined compared to controls (*CTL,* black bars) (n = 35 *CTL* rats, n = 14 *ID E0* rats,*p<0.05). **B**. The offspring from experimental group *ID E7* (blue hatched bars) exhibited significantly prolonged (P4-P1) interpeak latencies at all frequencies tested compared to *CTL* (black bars) (n = 35 *CTL rats*; n = 27 *ID E7 rats*, *p<0.05). **C**. The offspring born to experimental group *ID E14* (green hatched bars) had normal ABR (P4-P1) interpeak latencies compared to *CTL* (black bars) (n = 35 *CTL rats*; n = 29 *ID E14 rats*). The control and experimental dietary groups were composed of rat pups from 3 separate litters.

We also analyzed the P4-P1 interpeak latency differences to determine whether the impairment affects the entire brainstem auditory response in such a manner that changes observed at later peaks are even greater in magnitude than those detected through analysis of the P2-P1 interpeak latencies. As shown in **Figures 6** and **7**, ID animals showed a relatively larger increase than *CTL* when comparing the P4-P1 latencies (0.25–0.9 ms) to the P2-P1 latencies (0.5 ms - >1.5 ms). While all P4-P1 latencies, except one, were increased by over 1 ms in the *ID E0* group (**Figure 7A**), the increase in latency was less severe in the *ID E7* group. Nonetheless, even in this group, the alterations seen in the P4-P1 analysis were greater than in the P2-P1 analysis and also were significantly increased compared to control animals at all frequencies tested (**Figure 7B**). In contrast, and consistent with the measurements of P2-P1 changes, the *ID E14* animals did not show a significant difference in P4-P1 interpeak latencies compared to the control group (**Figure 7C**).

Taken together, our data show that dietary regimens that are not severe enough to cause a transition of maternal ID to severe IDA still disrupt fetal iron homeostasis as early as E15 and result in severe IDA in the offspring. We provide evidence showing that maternal iron restriction that occurs prior to the onset of pregnancy and during the first trimester has the most severe impact on the neural development of the offspring. Maternal ID during the second trimester still negatively affects the neural conduction velocity (measured by ABR) of the offspring but to a lesser extent. Our data further demonstrate that the development of neural impairments in the offspring is not dictated by the presence of IDA, low serum iron concentration or low birth weight, but is determined by the time window during which the dam received the iron-deficient diet.

## Discussion

The purpose of this study was to determine the impact of dietary maternal iron deficiency (ID) on fetal development, with the main focus on the identification of critical periods of gestation when the developing CNS is most vulnerable to maternal ID. The animal model we established was intended to mimic the extremely prevalent human condition of marginal maternal ID that occurs in an estimated 30 to 50 percent of pregnancies worldwide. After careful titration of the iron concentration in the diet, we were able to establish a model where the pregnant dam was rendered iron deficient but did not develop severe anemia during pregnancy.

The analysis of the most prolonged form of diet restriction, in which iron levels were already reduced prior to the onset of pregnancy led to some unexpected findings. For example, we found that despite the fact that the dams received the iron-deficient diet not only prior to conception but during the entire pregnancy, the dams did not develop severe anemia that had an impact on hematocrit levels, which remained in the normal range. However, maternal serum iron concentrations decreased continuously throughout pregnancy, confirming iron depletion. Upon more detailed examination of the maternal blood via HESKA Hema True Veterinary Hematology System, we observed minimal reductions in the measurement of mean corpuscular volume and hemoglobin compared to control values. Taken together, these results are consistent with ID and the absence of severe anemia.

This dietary model thus mimics a situation that would not cause any level of clinical concern in the human population, as the absence of severe anemia would not prompt any immediate intervention and is likely to remain unnoticed. We believe that this is an important aspect of our study as it underscores the need for monitoring the status of iron beyond the level of anemia.

Iron restriction to the dam two weeks prior to conception was associated with lower iron concentrations in embryonic tissues as early as embryonic day 15 (E15). The embryos maintained a very similar low iron level throughout gestation without an appreciable increase in serum iron concentrations suggesting that maternal iron stores, that apparently were not severely depleted, did not compensate for the continuously increased demand for iron by the embryo. Our observation seems to argue against the often proposed notion stating that the developing embryo is partly protected from the maternal ID through compensatory changes in the iron transport mechanisms of the placenta, which minimize the impact of ID in the fetus [68], [69], [70], [71]. However, our data is consistent with (i) recently published data, suggesting that gestational ID might have a restrictive effect on the constitution of adequate fetal iron stores, [72], [73] and with (ii) the observation by Naghii et al., indicating that fetal brain tissue can be iron depleted even in the absence of anemia in the pregnant mother [74]. Furthermore, studies from the Georgieff laboratory have shown that a transgenic model of hippocampus-specific iron deficiency without any signs of anemia also resulted in altered neuronal development as well as significant cognitive dysfunctions (reduced spatial recognition memory performance and procedural memory) [75], [76], [77]. Wu et al. have also shown that a short period of perinatal iron deficiency results in altered associative behavior in adult rats without anemia [78]. These studies support our notion that anemia is not necessarily a defining predictor of neural impairments that are associated with iron deficiency.

Our model of gestational ID was not only associated with persistently lower body weights as early as E15 but was also associated with functional impairments as determined by the Auditory Brainstem Responses (ABR) analysis of neural conduction velocity in the offspring. The increase in P2-P1 interpeak latencies without hearing threshold changes suggested that, at least in part, impaired myelination could be one explanation for our findings. While disruptions in myelination have been demonstrated in rodents with prenatal and lactational ID [43], [45], [79], [80], [81], [82], [83], [84] and hypomyelination is also recognized as a defining feature associated to perinatal ID in prematurely born infants [44], the greater increase in latency changes seen also by analyzing P4-P1 interpeak latencies (which reflects the entire brainstem) is consistent with findings in the literature suggesting that neuronal components could also be affected by iron deficiency.

As we established a functional criterion for the “diagnosis” of neural impairment that could clearly be associated with the maternal dietary intake, it was possible to design a timed dietary restriction study that cannot be performed in humans but is critical for understanding the window of vulnerability. We generated maternal dietary groups that were defined by the onset of iron restriction solely based on food source. Using the ABR test as a functional readout of the neural CNS integrity of the offspring, we determined that the maternal exposure to an iron-deficient diet either prior to conception, at the start of first trimester, or at the onset of second trimester had a significant negative impact on the ABR latency response in the offspring, placing the window of vulnerability for the fetus in the first two trimesters of gestation. Interestingly, the negative CNS effects on the fetus were more severe when the ID was initiated at the beginning of pregnancy compared to ID initiated prior to the onset of pregnancy. An important result of our research was that the offspring from pregnant rats receiving the ID diet at the beginning of the third trimester exhibited normal auditory nerve conduction velocities as reflected by ABR recordings despite IDA being present in all experimental rats at the time of testing. This result might shed some light on seemingly contradictory results found in humans; some investigators reported no alterations of ABR latencies in ID children with or without anemia [85], [86], while others found that IDA in 6 month old babies produced abnormally prolonged ABR latencies that persisted years after the children's iron status was corrected with iron therapy [43], [81]. Our study indicates that there is a precise window of vulnerability during which ID can affect the fetal CNS leading to postnatal neural impairments. The lack of defect seen in some studies could be a consequence of the insult occurring outside the window of vulnerability, rather than a perceived general insensitivity of newborns to iron restriction. Without the establishment of the maternal iron status during pregnancy or at least cord blood iron levels, it cannot be resolved whether the initiation of ID and the possible onset of a later apparent pathology happened *in utero*. Therefore, any conclusions about the importance and/or need of pre- and postnatal iron supplementation have to be made with caution.

**Figure 8:**
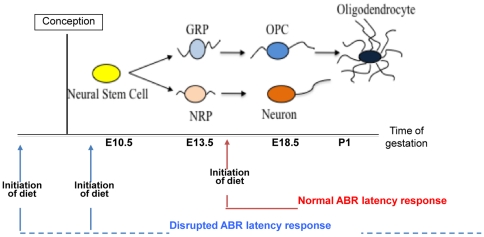
Proposed model displaying the impact of gestational iron restriction on characterized neural cell populations along a developmental time line. At embryonic day 10.5 (E10.5), multipotent neural stem cells can be isolated from the spinal cord. These cells give rise to glial restricted (GRP) and neuron restricted precursor cells (NRP) that can be isolated at around E13.5. Committed oligodendrocyte progenitor cells (OPC) are generated at around E18.5 and are thought to persist through early adulthood. The first mature myelin-producing oligodendrocytes arise around the day of birth (postnatal day 1). Blue arrows indicate the time of initiation of the iron-deficient diet that is associated with a disruption in the ABR conduction velocity at postnatal day 45 and mark the window of vulnerability. Red arrows represent the time of initiation of the iron-deficient diet that is not associated with impaired auditory nerve conduction.

The window of vulnerability, which we have defined in our studies, also offers an important clue for the underlying cellular impairment and seems to be consistent with our previous work on the study of cellular targets of ID [53]. As illustrated in **Figure 8**, ID during embryonic CNS development seems to affect neural precursor cell populations leading to an imbalance of specific precursor populations, which might be ultimately responsible for reduced oligodendrocyte numbers, abnormal myelination, and neural pathologies. We have shown previously that the differentiation and proliferation of embryonic glial precursor cells can be modulated by exogenous iron concentrations [87]. Interestingly, the timing of the generation, expansion and differentiation of these precursor cells coincides with the window of vulnerability we have defined in our diet regimens. In the rat embryo, gliogenesis starts around day E13.5 with the generation of embryonic glial restricted precursor cells (GRPs), which shortly before birth give rise to oligodendrocyte precursors (OPCs) that persist postnatally [88]. Even slight alterations of proliferation and/or of differentiation abilities of precursor cells (GRPs and OPCs) occurring before the time of oligodendrocyte generation could translate into a myelination defect. The notion of early embryonic cells being especially responsive to suboptimal iron levels is also supported by studies from Badarocco et al., whose interesting experiments using intracranial apotransferrin injections suggest that iron may affect oligodendrocyte development at early stages of embryogenesis rather than during late development [89]. The defects that are associated with iron deficiency are, however, unlikely to be restricted to the glial population. As shown in **Figure 8**, neuronal progenitor cells develop in a similar time window and could equally well be targets of iron deficiency. The cognitive impairment seen in the offspring is most likely a combination of an impact on both neuronal and glial embryonic progenitor cell pools.

The absence of a functional defect in late onset ID suggests that, if the diet is introduced at embryonic day 14 (red arrow) and maintained, the time point of iron depletion is past the period when the most vulnerable embryonic populations expand and generate their progeny. Under these conditions, the impact of ID on glial cell generation and/or neuronal maturation does not seem to be severe enough to result in detectable auditory dysfunction.

It should be pointed out that despite the lack of an ABR latency defect in our study at the late gestational time point (*ID E14*), we cannot exclude the presence of other abnormalities. For instance, a number of studies in rodent and primate models of late gestational and postnatal ID reported significant and persistent behavioral abnormalities and neurochemical disturbances [19], [90], [91], [92], [93], [94], [95], [96], [97]. As development occurs along a temporal continuum, it would be expected that deficiencies occurring at different times cause different types of outcomes.

The timed maternal iron restriction coupled with a persistently deficient diet used in our model, led to another surprising finding that sheds some new light on the role of anemia in iron deficiency. Due to the associated fatigue, muscle weakness, and dyspnea during exertion, anemia is considered a confounding factor in various behavioral tests and raises criticism on the accuracy of the results and their interpretation. The ABR electrophysiological test we employed in the evaluation of neurodevelopmental impairments induced by ID seems not to be influenced by anemia. As shown in **Figure 5A**, all offspring of our different feeding groups developed clinically relevant anemia, yet only three out of four groups showed functional impairment in ABR patterns. This observation strongly suggests that anemia is not a determinant factor of pathological ABR patterns recorded in iron deficient animals and allows dissociation of the impairment caused by ID from effects that might be primarily due to anemia. As ABR analysis can be conducted in multiple species, including humans, this type of analysis may provide a particularly useful noninvasive tool for examining the effects of micronutrient deficiencies on CNS development.

While IDA was not required to cause ABR defects, the occurrence of IDA did, however, severely impact the survival rate of the offspring. The litter survival rate was approximately 75% in the four experimental groups compared to the control groups. This decrease in survival rate may lead to an unintentional sample population bias of “survivors” at P45 when the functional measurements are conducted. While we cannot completely rule out that the surviving animals are on some level more robust that their littermates, it is important to note that we cannot correlate survival with absence or presence of ABR defects. The *ID -2wk, ID E0* and *ID E7* groups had as many animals that died during the experiments as the *ID E14* group yet none of the surviving animals in the last group showed an ABR defect, while all the animals in the other three groups showed an ABR defect of various severities.

Another novel observation in our study is the time-specific presence of thermoregulatory deficits in the offspring from mothers who were iron restricted two weeks prior to conception. The three other feeding groups did not develop such defects. As ID has sometimes been linked to poor thyroid function [98], which in turn can result in a thermoregulatory dysfunction [99], [100], [101], [102], [103], the decreased core body temperature in the most severe feeding regimen (*ID -2wks)* might involve a thyroid component. However, based on our data, we do not think that thyroid involvement is critical for the development of the ABR dysfunction, as the *ID E0* and the *ID E7* groups have significant ABR dysfunctions but normal core body temperatures.

In summary, the analysis of developmental defects that are associated with exposure to iron restriction during different phases of fetal development emphasizes the influence of the precise window of vulnerability and the associated duration and magnitude of ID that is exerted on the developing fetal CNS. The prolonged absolute and interpeak latencies revealed by the comprehensive ABR analysis in ID animals could have important clinical implications. If ID at an early age causes hypomyelination or delayed neural maturation along the auditory pathway altering the properties of the auditory message and its transmission through the brainstem structures, it is possible that such a dysfunction could have functional significance for language acquisition and the development of other higher cognitive and emotional functions.

In lieu of this information, our data not only reiterates the importance of iron supplementation as part of prenatal and gestational IDA therapeutic regimen but also as a preventive strategy. Identifying the windows of vulnerability when specifically vulnerable cell populations are generated during embryonic development will be critical in delineating the restricted window of therapeutic opportunity when iron supplementation could correct or prevent the functional deficit.

## Materials and Methods

### Animals and dietary treatment

Adult female and male Sprague-Dawley rats (10–12 weeks of age) were purchased from Charles River Laboratories (Wilmington, MA). All animals were housed individually under controlled environmental conditions (0600–1800 h light cycle, 23°C and 32% humidity) and were provided free access to food and water.

For the time mated pregnancies, the female/male pair was housed in the same cage for 24–48 hours or until the detection of vaginal plug. There are no data to support a notion of decreased fertility in any of the iron deficient (ID) diet treated groups. The litter size was comparable to controls and the newborn pups appeared healthy although their overall size is smaller in the *ID -2wk* and *ID E0* groups. We did, however, observe a decreased survival rate in all iron deficiency groups. By postnatal day 45, on average 10–20% of the offspring died with the most severe losses in the *ID -2wk* group. The lower survival rate was most likely a result of the severe anemia experienced by the offspring.

### Determination of gestational age

For the time-mated pregnancies, the first day of gestation was determined by the appearance of vaginal plug. For exact timing of the gestational age, the crown-to-rump length (CRL) of the embryo was determined. The measurement of CRL, using a standard millimeter scale, was taken from the vertex to the rump with care taken to avoid pressure that would distort the natural curvature of the embryos. Embryonic size based on CRL in addition to vaginal plug detection allowed for a more accurate evaluation of gestational age.

### Experimental design (dietary regimens)

The feeding protocols used in our study are depicted in **Figure 4**. The experimental animals were divided in four groups of specific dietary regimens, using the same iron-deficient diet containing 2–6 µg Fe/g of food (rodent TD. 80396 ID Diet – Harlan Teklad Custom Research Diets).

The first diet regimen was designed to generate embryonic ID in early pregnancy. The dams were started on the iron-deficient diet two weeks before conception and the pups born to these dams represented the experimental group *“* ***ID -2wks”*** *.*

The next group of experimental animals was fed the iron-deficient diet on the day of conception or appearance of vaginal plug, designated **“** ***ID E0”*** *.*

The third experimental group of animals, designated *“* ***ID E7”*** **,** comprised of rat pups born to dams exposed to the iron-deficient diet from the seventh gestational day onwards.

The rat pups born to dams feeding on the iron-deficient diet from gestational day 14 onwards were assigned to the fourth dietary group of rats, labeled *“* ***ID E14”*** *.*

As our control **(** ***CTL)***, we used age-matched rats born to dams that were maintained on control iron diet throughout the duration of the experiment. The control diet contained about 240 µg Fe/g of food (Adjusted Iron Diet (240) – TD.05656 from Harlan Teklad Custom Research Diets). All other components of the diet, composition of nutrients and caloric values were identical to the ID diet except the concentration of iron. All control and experimental animals were given deionized-distilled water through glass sipper tubes.

All groups received the assigned dietary treatment (iron-deficient and control diets) during lactation and after weaning until postnatal day 45, when the animals were subjected to ABR and DPOAE testing as well as evaluation of body weight, core body temperature, and iron status. Animal procedures were approved by the University Committee of Animal Resources at the University of Rochester, New York.

### Hematocrit and serum iron level measurement

The animals were anesthetized with ketamine (100 mg/kg i.p.) and blood samples for hematocrit measurement were collected in heparinized microcapillary tubes (VWR, West Chester, PA) from the right cardiac atrium before the rats were perfused. Tubes were centrifuged for 15 minutes at room temperature in a high speed hematocrit centrifuge (IEC MB Microhematocrit Centrifuge). The Hematocrit value was calculated as the length of red blood cell column per total blood sample column length and expressed as a percentage.

For a more detailed hematological analysis including hematocrit (Ht) values, hemoglobin (Hb) concentrations, red blood cell (RBC) counts, and mean corpuscular volumes (MCV), we analyzed the fresh blood samples, collected in EDTA-coated tubes, using HESKA Hema True Veterinary Hematology System.

Whole blood aliquots for serum iron determination were allowed to clot, and then were centrifuged at 10,000 x g at 4°C for 15 minutes to separate cells from sera. Serum samples were isolated then frozen and stored at −80°C until analysis for iron concentrations by atomic absorption spectrophotometry (AAS).

### Tissue iron measurement using Atomic Absorption Spectroscopy (AAS)

After anesthesia with ketamine (100 mg/kg i.p.) and blood collection for hematocrit and serum iron determination, the animals were transcardially perfused with11.2 µg/ml heparin in phosphate-buffered saline via the left ventricle to remove blood from organs. The embryos or CNS tissues were immediately dissected, washed in distilled deionized water, vacuum dried, weighed, and stored in iron-free teflon vials at −80°C until analyzed as previously described. Frozen embryonic and postnatal CNS tissues were wet-digested in ultra-pure nitric acid and analyzed for iron concentration using the graphite furnace atomic absorption spectrophotometer. Standards, blanks, and standard curves were used as previously specified [104], [105], [106].

### Core body temperature measurement

All experimental ID and control rats were subjected to core body temperature measurement by using a RET-2 rectal probe and TH-5 Thermalert Monitoring Thermometer (Physitemp). The core body temperature was consistently recorded for all groups of animals at six o'clock in the evening of the day of auditory testing.

### Auditory Brainstem Responses (ABR) recording and analysis

Control (*CTL*) and iron deficient (ID) dietary groups of rats (*ID -2wks*, *ID E0*, *ID E7*, and *ID E14* groups) were ABR tested on postnatal day 40–45. For the test, rats were anesthetized with acepromazine (1 mg/kg i.p.) and ketamine (80–100 mg/kg i.p.). Needle electrodes were inserted at vertex and pinna, with a ground near the tail. ABR potentials were evoked with click tone pips at 5 log-spaced frequencies from 5.6 to 24.4 kHz at 70 dB SPL. The response was amplified (10,000x) and 2,048 responses were averaged with using software and high-frequency speakers from Intelligent Hearing Systems (Smart EP, Miami, FL). To compare the change of latencies, the ABR peaks and troughs were identified by a semi-automated tool and verified by a trained observer by visual inspection of recorded wave forms. Latencies were calculated from the onset of the stimulus, and the interpeak latencies of wave II-I (P2-P1), wave III-I (P3-P1), and wave IV-I (P4-P1) were computed. The change of interpeak latencies was then calculated.

### Distortion Product of Otoacoustic Emissions (DPOAE) acquisition

The function of the outer hair cells in the ear was analyzed by measuring DPOAEs with secondary tones (f2) at 5 log-spaced frequencies from 5.6 to 24.4 kHz. DPOAE testing was performed with f2/f1 = 1.2 and unequal primaries (L1 = 10dB+L2) at 70 dB and 60 dB SPL using high-frequency speakers and software from Intelligent Hearing Systems (SmartOAE) and high-frequency microphones (ER10B+) from Etymotic Research (Elk Grove Village, IL). Cochlear function was assessed for all animals at approximately 40–45 days of age and rats were sedated with ketamine (80–100 mg/kg i.p.) and acepromazine (1 mg/kg i.p.).

### Statistical analyses

“n” represents the number of animals in any group analyzed and has a value ≥ 3. Regardless the sample size (n value), each control and experimental group was composed of animals from at least 3 separate litters. Statistical analyses were performed with the two-tailed Student's t-test and conducted comparing nutritionally ID to control rats, under the same conditions. A p-value less than 0.05 was considered statistically significant. Comparison of mean values of ABR interpeak latencies between different dietary groups and controls was performed with analysis of variance (ANOVA) using a Bonferroni posttest analysis and significance was defined as a p value less than 0.05.

## References

[1] Stoltzfus RJ (2003). Iron deficiency: global prevalence and consequences.. Food Nutr Bull.

[2] Baynes RD, Cook JD (1996). Current issues in iron deficiency.. Curr Opin Hematol.

[3] Beard J (2003). Iron deficiency alters brain development and functioning.. J Nutr.

[4] Conrad ME, Umbreit JN (2001). Iron absorption: relative importance of iron transport pathways.. Am J Hematol.

[5] Conrad ME, Umbreit JN, Moore EG, Hainsworth LN, Porubcin M (2000). Separate pathways for cellular uptake of ferric and ferrous iron.. Am J Physiol Gastrointest Liver Physiol.

[6] Casanueva E, Viteri FE (2003). Iron and oxidative stress in pregnancy.. J Nutr.

[7] Melamed N, Ben-Haroush A, Kaplan B, Yogev Y (2007). Iron supplementation in pregnancy–does the preparation matter?. Arch Gynecol Obstet.

[8] Nguyen P, Nava-Ocampo A, Levy A, O'Connor DL, Einarson TR (2008). Effect of iron content on the tolerability of prenatal multivitamins in pregnancy.. BMC Pregnancy Childbirth.

[9] Siega-Riz AM, Hartzema AG, Turnbull C, Thorp J, McDonald T (2006). The effects of prophylactic iron given in prenatal supplements on iron status and birth outcomes: a randomized controlled trial.. Am J Obstet Gynecol.

[10] deRegnier RA, Long JD, Georgieff MK, Nelson CA (2007). Using event-related potentials to study perinatal nutrition and brain development in infants of diabetic mothers.. Dev Neuropsychol.

[11] Georgieff MK (2006). The effect of maternal diabetes during pregnancy on the neurodevelopment of offspring.. Minn Med.

[12] Love AL, Billett HH (2008). Obesity, bariatric surgery, and iron deficiency: true, true, true and related.. Am J Hematol.

[13] Xanthakos SA (2009). Nutritional deficiencies in obesity and after bariatric surgery.. Pediatr Clin North Am.

[14] Banhidy F, Acs N, Puho EH, Czeizel AE (2010). Iron deficiency anemia: Pregnancy outcomes with or without iron supplementation.. Nutrition.

[15] El Guindi W, Pronost J, Carles G, Largeaud M, El Gareh N (2004). Severe maternal anemia and pregnancy outcome.. J Gynecol Obstet Biol Reprod (Paris).

[16] Lee HS, Kim MS, Kim MH, Kim YJ, Kim WY (2006). Iron status and its association with pregnancy outcome in Korean pregnant women.. Eur J Clin Nutr.

[17] Scholl TO (2005). Iron status during pregnancy: setting the stage for mother and infant.. Am J Clin Nutr.

[18] Singla PN, Tyagi M, Kumar A, Dash D, Shankar R (1997). Fetal growth in maternal anaemia.. J Trop Pediatr.

[19] Beard J (2007). Recent evidence from human and animal studies regarding iron status and infant development.. J Nutr.

[20] Beard JL, Connor JD, Jones BC (1993). Brain iron: location and function.. Prog Food Nutr Sci.

[21] Beard JL, Hendricks MK, Perez EM, Murray-Kolb LE, Berg A (2005). Maternal iron deficiency anemia affects postpartum emotions and cognition.. J Nutr.

[22] Corapci F, Radan AE, Lozoff B (2006). Iron deficiency in infancy and mother-child interaction at 5 years.. J Dev Behav Pediatr.

[23] Eden AN (2005). Iron deficiency and impaired cognition in toddlers: an underestimated and undertreated problem.. Paediatr Drugs.

[24] Grantham-McGregor S, Ani C (2001). A review of studies on the effect of iron deficiency on cognitive development in children.. J Nutr.

[25] Halterman JS, Kaczorowski JM, Aligne CA, Auinger P, Szilagyi PG (2001). Iron deficiency and cognitive achievement among school-aged children and adolescents in the United States.. Pediatrics.

[26] Lozoff B (1989). Iron and learning potential in childhood.. Bull N Y Acad Med.

[27] Lozoff B (2000). Perinatal iron deficiency and the developing brain.. Pediatr Res.

[28] Lozoff B, Beard J, Connor J, Barbara F, Georgieff M (2006). Long-lasting neural and behavioral effects of iron deficiency in infancy.. Nutr Rev.

[29] Lozoff B, Brittenham GM (1986). Behavioral aspects of iron deficiency.. Prog Hematol.

[30] Lozoff B, Brittenham GM (1987). Behavioral alterations in iron deficiency.. Hematol Oncol Clin North Am.

[31] Lozoff B, Brittenham GM, Wolf AW, McClish DK, Kuhnert PM (1987). Iron deficiency anemia and iron therapy effects on infant developmental test performance.. Pediatrics.

[32] Lozoff B, Clark KM, Jing Y, Armony-Sivan R, Angelilli ML (2008). Dose-response relationships between iron deficiency with or without anemia and infant social-emotional behavior.. J Pediatr.

[33] Lukowski AF, Koss M, Burden MJ, Jonides J, Nelson CA (2010). Iron deficiency in infancy and neurocognitive functioning at 19 years: evidence of long-term deficits in executive function and recognition memory.. Nutr Neurosci.

[34] Wachs TD, Pollitt E, Cueto S, Jacoby E, Creed-Kanashiro H (2005). Relation of neonatal iron status to individual variability in neonatal temperament.. Dev Psychobiol.

[35] Walter T (1994). Effect of iron-deficiency anaemia on cognitive skills in infancy and childhood.. Baillieres Clin Haematol.

[36] Brigham D, Beard J (1996). Iron and thermoregulation: a review.. Crit Rev Food Sci Nutr.

[37] Ece A, Yigitoglu MR, Vurgun N, Guven H, Iscan A (1999). Serum lipid and lipoprotein profile in children with iron deficiency anemia.. Pediatr Int.

[38] Hartfield DS, Lowry NJ, Keene DL, Yager JY (1997). Iron deficiency: a cause of stroke in infants and children.. Pediatr Neurol.

[39] Hartfield DS, Tan J, Yager JY, Rosychuk RJ, Spady D (2009). The association between iron deficiency and febrile seizures in childhood.. Clin Pediatr (Phila).

[40] Angulo-Kinzler RM, Peirano P, Lin E, Algarin C, Garrido M (2002). Twenty-four-hour motor activity in human infants with and without iron deficiency anemia.. Early Hum Dev.

[41] Angulo-Kinzler RM, Peirano P, Lin E, Garrido M, Lozoff B (2002). Spontaneous motor activity in human infants with iron-deficiency anemia.. Early Hum Dev.

[42] Shafir T, Angulo-Barroso R, Jing Y, Angelilli ML, Jacobson SW (2008). Iron deficiency and infant motor development.. Early Hum Dev.

[43] Algarin C, Peirano P, Garrido M, Pizarro F, Lozoff B (2003). Iron deficiency anemia in infancy: long-lasting effects on auditory and visual system functioning.. Pediatr Res.

[44] Amin SB, Orlando M, Eddins A, MacDonald M, Monczynski C (2009). In utero iron status and auditory neural maturation in premature infants as evaluated by auditory brainstem response.. J Pediatr.

[45] Cankaya H, Oner AF, Egeli E, Caksen H, Uner A (2003). Auditory brainstem response in children with iron deficiency anemia.. Acta Paediatr Taiwan.

[46] Lozoff B, De Andraca I, Castillo M, Smith JB, Walter T (2003). Behavioral and developmental effects of preventing iron-deficiency anemia in healthy full-term infants.. Pediatrics.

[47] De Pee S, Bloem MW, Sari M, Kiess L, Yip R (2002). The high prevalence of low hemoglobin concentration among Indonesian infants aged 3-5 months is related to maternal anemia.. J Nutr.

[48] Emamghorashi F, Heidari T (2004). Iron status of babies born to iron-deficient anaemic mothers in an Iranian hospital.. East Mediterr Health J.

[49] Georgieff MK, Wewerka SW, Nelson CA, Deregnier RA (2002). Iron status at 9 months of infants with low iron stores at birth.. J Pediatr.

[50] Fairbanks VF (1994). Blue gods, blue oil, and blue people.. Mayo Clin Proc.

[51] Florentino RF, Villavieja GM, Boquecosa JP, Bacos FF (1992). Nutrition situation in metro Manila.. Southeast Asian J Trop Med Public Health.

[52] Looker AC, Dallman PR, Carroll MD, Gunter EW, Johnson CL (1997). Prevalence of iron deficiency in the United States.. Jama.

[53] Morath DJ, Mayer-Proschel M (2002). Iron deficiency during embryogenesis and consequences for oligodendrocyte generation in vivo.. Dev Neurosci.

[54] Cutmore TR (2004). The use of wavelets for auditory brain-stem analysis: advocacy and precautions.. Clin Neurophysiol.

[55] Hall JW, Rupp KA (1997). Auditory brainstem response: recent developments in recording and analysis.. Adv Otorhinolaryngol.

[56] Legatt AD (2002). Mechanisms of intraoperative brainstem auditory evoked potential changes.. J Clin Neurophysiol.

[57] Shaw NA (1995). The temporal relationship between the brainstem and primary cortical auditory evoked potentials.. Prog Neurobiol.

[58] Ito T, Tokuriki M, Shibamori Y, Saito T, Nojyo Y (2004). Cochlear nerve demyelination causes prolongation of wave I latency in ABR of the myelin deficient (md) rat.. Hear Res.

[59] Knipper M, Bandtlow C, Gestwa L, Kopschall I, Rohbock K (1998). Thyroid hormone affects Schwann cell and oligodendrocyte gene expression at the glial transition zone of the VIIIth nerve prior to cochlea function.. Development.

[60] Church MW, Jen KL, Jackson DA, Adams BR, Hotra JW (2009). Abnormal neurological responses in young adult offspring caused by excess omega-3 fatty acid (fish oil) consumption by the mother during pregnancy and lactation.. Neurotoxicol Teratol.

[61] Noben-Trauth K, Latoche JR, Neely HR, Bennett B (2010). Phenotype and genetics of progressive sensorineural hearing loss (Snhl1) in the LXS set of recombinant inbred strains of mice.. PLoS One.

[62] Ponton CW, Moore JK, Eggermont JJ (1996). Auditory brain stem response generation by parallel pathways: differential maturation of axonal conduction time and synaptic transmission.. Ear Hear.

[63] Wang Y, Manis PB (2005). Synaptic transmission at the cochlear nucleus endbulb synapse during age-related hearing loss in mice.. J Neurophysiol.

[64] Maison SF, Casanova E, Holstein GR, Bettler B, Liberman MC (2009). Loss of GABAB receptors in cochlear neurons: threshold elevation suggests modulation of outer hair cell function by type II afferent fibers.. J Assoc Res Otolaryngol.

[65] Park HJ, Kim HJ, Bae GS, Seo SW, Kim DY (2009). Selective GSK-3beta inhibitors attenuate the cisplatin-induced cytotoxicity of auditory cells.. Hear Res.

[66] Tanaka C, Chen GD, Hu BH, Chi LH, Li M (2009). The effects of acoustic environment after traumatic noise exposure on hearing and outer hair cells.. Hear Res.

[67] Wu HP, Hsu CJ, Cheng TJ, Guo YL (2010). N-acetylcysteine attenuates noise-induced permanent hearing loss in diabetic rats.. Hear Res.

[68] Chong WS, Kwan PC, Chan LY, Chiu PY, Cheung TK (2005). Expression of divalent metal transporter 1 (DMT1) isoforms in first trimester human placenta and embryonic tissues.. Hum Reprod.

[69] Dallman PR (1986). Iron deficiency in the weanling: a nutritional problem on the way to resolution.. Acta Paediatr Scand Suppl.

[70] Gambling L, Danzeisen R, Gair S, Lea RG, Charania Z (2001). Effect of iron deficiency on placental transfer of iron and expression of iron transport proteins in vivo and in vitro.. Biochem J.

[71] Munoz C, Olivares M, Schlesinger L, Lopez M, Letelier A (1994). Increased in vitro tumour necrosis factor-alpha production in iron deficiency anemia.. Eur Cytokine Netw.

[72] Jaime-Perez JC, Herrera-Garza JL, Gomez-Almaguer D (2005). Sub-optimal fetal iron acquisition under a maternal environment.. Arch Med Res.

[73] Sweet DG, Savage G, Tubman TR, Lappin TR, Halliday HL (2001). Study of maternal influences on fetal iron status at term using cord blood transferrin receptors.. Arch Dis Child Fetal Neonatal Ed.

[74] Naghii MR, Fouladi AI (2006). Correct assessment of iron depletion and iron deficiency anemia.. Nutr Health.

[75] Brunette KE, Tran PV, Wobken JD, Carlson ES, Georgieff MK (2010). Gestational and neonatal iron deficiency alters apical dendrite structure of CA1 pyramidal neurons in adult rat hippocampus.. Dev Neurosci.

[76] Carlson ES, Fretham SJ, Unger E, O'Connor M, Petryk A (2010). Hippocampus specific iron deficiency alters competition and cooperation between developing memory systems.. J Neurodev Disord.

[77] Carlson ES, Tkac I, Magid R, O'Connor MB, Andrews NC (2009). Iron is essential for neuron development and memory function in mouse hippocampus.. J Nutr.

[78] Wu L-l, Zhang L, Shao J, Qin Y-f, Yang R-w (2008). Effect of perinatal iron deficiency on myelination and associated behaviors in rat pups.. Behavioural Brain Research.

[79] Beard JL, Erikson KM, Jones BC (2002). Neurobehavioral analysis of developmental iron deficiency in rats.. Behav Brain Res.

[80] Erikson KM, Pinero DJ, Connor JR, Beard JL (1997). Regional brain iron, ferritin and transferrin concentrations during iron deficiency and iron repletion in developing rats.. J Nutr.

[81] Roncagliolo M, Garrido M, Walter T, Peirano P, Lozoff B (1998). Evidence of altered central nervous system development in infants with iron deficiency anemia at 6 mo: delayed maturation of auditory brainstem responses.. Am J Clin Nutr.

[82] Unger EL, Paul T, Murray-Kolb LE, Felt B, Jones BC (2007). Early iron deficiency alters sensorimotor development and brain monoamines in rats.. J Nutr.

[83] Zhou B, Sun A, Wang S, Ma Y, Shen J (2001). [The effects of iron supplementation on auditory brain-stem response with iron deficiency anemia in rats].. Lin Chuang Er Bi Yan Hou Ke Za Zhi.

[84] Zhou GD, Randerath E, Randerath K (2001). Effects of dietary transition metals on oxidative DNA lesions in neonatal rats.. Mutat Res.

[85] Kurekci AE, Sarici SU, Karaoglu A, Ulas UH, Atay AA (2006). Effects of iron deficiency versus iron deficiency anemia on brainstem auditory evoked potentials in infancy.. Turk J Pediatr.

[86] Sarici SU, Serdar MA, Dundaroz MR, Unay B, Akin R (2001). Brainstem auditory-evoked potentials in iron-deficiency anemia.. Pediatr Neurol.

[87] Morath DJ, Mayer-Proschel M (2001). Iron modulates the differentiation of a distinct population of glial precursor cells into oligodendrocytes.. Dev Biol.

[88] Gregori N, Proschel C, Noble M, Mayer-Proschel M (2002). The tripotential glial-restricted precursor (GRP) cell and glial development in the spinal cord: generation of bipotential oligodendrocyte-type-2 astrocyte progenitor cells and dorsal-ventral differences in GRP cell function.. J Neurosci.

[89] Badaracco ME, Siri MVR, Pasquini JM (2010). Oligodendrogenesis: The role of iron.. Biofactors.

[90] Bourque SL, Iqbal U, Reynolds JN, Adams MA, Nakatsu K (2008). Perinatal iron deficiency affects locomotor behavior and water maze performance in adult male and female rats.. J Nutr.

[91] Burhans MS, Dailey C, Beard Z, Wiesinger J, Murray-Kolb L (2005). Iron deficiency: differential effects on monoamine transporters.. Nutr Neurosci.

[92] Felt BT, Beard JL, Schallert T, Shao J, Aldridge JW (2006). Persistent neurochemical and behavioral abnormalities in adulthood despite early iron supplementation for perinatal iron deficiency anemia in rats.. Behav Brain Res.

[93] Gambling L, Andersen HS, Czopek A, Wojciak R, Krejpcio Z (2004). Effect of timing of iron supplementation on maternal and neonatal growth and iron status of iron-deficient pregnant rats.. J Physiol.

[94] Kwik-Uribe CL, Gietzen D, German JB, Golub MS, Keen CL (2000). Chronic marginal iron intakes during early development in mice result in persistent changes in dopamine metabolism and myelin composition.. J Nutr.

[95] Kwik-Uribe CL, Golub MS, Keen CL (2000). Chronic marginal iron intakes during early development in mice alter brain iron concentrations and behavior despite postnatal iron supplementation.. J Nutr.

[96] Reeves PG, DeMars LC (2006). Signs of iron deficiency in copper-deficient rats are not affected by iron supplements administered by diet or by injection.. J Nutr Biochem.

[97] Schmidt AT, Waldow KJ, Grove WM, Salinas JA, Georgieff MK (2007). Dissociating the long-term effects of fetal/neonatal iron deficiency on three types of learning in the rat.. Behav Neurosci.

[98] Zimmermann MB, Kohrle J (2002). The impact of iron and selenium deficiencies on iodine and thyroid metabolism: biochemistry and relevance to public health.. Thyroid.

[99] Beard J, Finch CA, Green WL (1982). Interactions of iron deficiency, anemia, and thyroid hormone levels in response of rats to cold exposure.. Life Sci.

[100] Beard J, Green W, Miller L, Finch C (1984). Effect of iron-deficiency anemia on hormone levels and thermoregulation during cold exposure.. Am J Physiol.

[101] Beard J, Tobin B, Green W (1989). Evidence for thyroid hormone deficiency in iron-deficient anemic rats.. J Nutr.

[102] Dillman E, Gale C, Green W, Johnson DG, Mackler B (1980). Hypothermia in iron deficiency due to altered triiodothyronine metabolism.. Am J Physiol.

[103] Tang F, Wong TM, Loh TT (1988). Effects of cold exposure or TRH on the serum TSH levels in the iron-deficient rat.. Horm Metab Res.

[104] Focht SJ, Snyder BS, Beard JL, Van Gelder W, Williams LR (1997). Regional distribution of iron, transferrin, ferritin, and oxidatively-modified proteins in young and aged Fischer 344 rat brains.. Neuroscience.

[105] van Gelder W, Huijskes-Heins MI, Hukshorn CJ, de Jeu-Jaspars CM, van Noort WL (1995). Isolation, purification and characterization of porcine serum transferrin and hemopexin.. Comp Biochem Physiol B Biochem Mol Biol.

[106] van Gelder W, Huijskes-Heins MI, van Dijk JP, Cleton-Soeteman MI, van Eijk HG (1995). Quantification of different transferrin receptor pools in primary cultures of porcine blood-brain barrier endothelial cells.. J Neurochem.

